# Investigation of Rice Yields and Critical N Losses from Paddy Soil under Different N Fertilization Rates with Iron Application

**DOI:** 10.3390/ijerph19148707

**Published:** 2022-07-17

**Authors:** Weishou Shen, Yaou Long, Zijian Qiu, Nan Gao, Yoko Masuda, Hideomi Itoh, Hirotomo Ohba, Yutaka Shiratori, Adharsh Rajasekar, Keishi Senoo

**Affiliations:** 1 Jiangsu Key Laboratory of Atmospheric Environment Monitoring and Pollution Control, Collaborative Innovation Center of Atmospheric Environment and Equipment Technology, School of Environmental Science and Engineering, Nanjing University of Information Science and Technology, Nanjing 210044, China; wsshen@nuist.edu.cn (W.S.); L410169283@hotmail.com (Y.L.); 20211900010@nuist.edu.cn (Z.Q.); 870031@nuist.edu.cn (A.R.); 2 National Engineering Research Center for Biotechnology, School of Biological and Pharmaceutical Engineering, Nanjing Tech University, Nanjing 211816, China; 3 Department of Applied Biological Chemistry, Graduate School of Agricultural and Life Sciences, The University of Tokyo, Tokyo 113-8657, Japan; ygigico@gmail.com; 4 Microbial Ecology and Technology Research Group, Bioproduction Research Institute, National Institute of Advanced Industrial Science and Technology (AIST), Toyohira-ku, Sapporo 062-8517, Japan; hideomi-itou@aist.go.jp; 5 Agro-Environment Research Division, Niigata Agricultural Research Institute, Nagaoka 940-0826, Japan; ohba.hirotomo@pref.niigata.lg.jp (H.O.); shiratori.yutaka@pref.niigata.lg.jp (Y.S.); 6 Collaborative Research Institute for Innovative Microbiology, The University of Tokyo, Tokyo 113-8657, Japan

**Keywords:** ammonia volatilization, iron-reducing bacteria, nitrogen fixation, NO_3_^−^ leaching, nitrous oxide emissions

## Abstract

The application of iron powder stimulated the growth of iron-reducing bacteria as a respiratory substrate and enhanced their nitrogen (N)-fixing activity in flooded paddy soils. High N fertilization (urea) in the flooded paddy soils has caused adverse environmental impacts such as ammonia (NH_3_) volatilization, nitrous oxide (N_2_O) emissions, and nitrate (NO_3_^−^) leaching. This study aims to investigate the effects of N fertilization rates in combination with an iron amendment on rice yields and N losses from flooded paddy fields. We performed a 2-year field plot experiment with traditional rice–wheat rotation in China’s Yangtze River Delta. The investigation consisted of seven treatments, including 100%, 80%, 60%, and 0% of the conventional N (urea and commercial organic manure) fertilization rate, and 80%, 60%, and 0% of the conventional N with the iron powder (≥99% purity) amendment. The rice yields decreased with a reduction in the conventional N fertilization rate, whereas they were comparable after the iron application under the 80% and 60% conventional N rate. The critical N losses, including NH_3_ volatilization, N_2_O emissions, and NO_3_^−^ and NH_4_^+^ leaching, generally decreased with a reduction in the conventional N fertilization rate. These N losses were significantly greater after the iron amendment compared with the non-amended treatments under the 80% and 60% conventional N fertilization rate in the first rice-growing season. However, it was comparable between the iron-amended and the non-amended treatments in the second season. Furthermore, NO_3_^−^ leaching was the most significant N loss throughout the two rice seasons, followed by NH_3_ volatilization. The iron amendment significantly increased soil Fe^2+^ content compared with the non-amended treatments irrespective of N fertilization, suggesting the reduction of amended iron by iron-reducing bacteria and their simultaneous N fixation. A combination of the iron application with 60–80% of the conventional N fertilization rate could maintain rice yields similar to the conventional N fertilization rate while reducing the critical N losses in the flooded paddy field tested in this study. Our study leads to the establishment of novel and practical rice cultivation, which is a step towards the development of green agriculture.

## 1. Introduction

Rice (*Oryza sativa* L.) is one of the most important cereal crops planted worldwide [1,2]. The application of synthetic fertilizer, particularly N fertilizer, is an important and effective way to increase rice yield and meet the global demand for food. The growing demand for cereal crops keeps increasing the use of N fertilizer due to the world’s growing population [3,4,5]. However, synthetic fertilizer production is a high energy consumption process, accounting for about 1.2% of global energy consumption, of which 93% is used for the production of N fertilizer [6,7]. Moreover, a large amount of N fertilizer is used inefficiently and, thus, has decreased N use efficiency and caused critical N loss such as NH_3_ volatilization, N_2_O emissions, and NO_3_^−^ leaching [8,9,10,11]. The N losses have aggravated environmental pollution, such as air haze, global warming, water eutrophication, and excessive NO_3_^−^ in groundwater, thereby threatening the health and safety of human beings and the wider environment [8,12,13,14].

China’s rice yield and planting area rank first and second worldwide, respectively, to effectively ensure national food security. From 1997–2006, the Chinese national average N application rate for rice increased by two-fold (145 kg N hm^−2^ in 1997 to 300 kg N hm^−2^ in 2006), which is significantly higher than the global average. The average N fertilization rate is ca. 180 kg N hm^−2^ in flooded paddy fields across China, which is 75% higher than that of global flooded paddy fields and two times that of flooded paddy fields across Japan [8,15,16]. Consequently, the environmental issues caused by the extensive or even excessive use of N fertilizer have aroused public concern. The Chinese government launched a national strategy for reducing synthetic fertilizer use in 2015 [17]. Measures for developing green agriculture are greatly encouraged to enhance crop yields and reduce environmental pollution.

Biological N fixation (BNF) refers to the conversion of N_2_ to NH_3_ under the action of nitrogenase produced by N-fixing bacteria at an average temperature and pressure [18]. BNF is receiving increasing attention worldwide since it consumes almost no energy from fossil fuels and does not cause N pollution [19,20,21,22]. Previous studies have shown that BNF can contribute significantly to the N fertility of soil without supplying synthetic fertilizers for thousands of years to the flooded paddy soils in Asia [23,24]. For example, using the ^15^N-labeled soil method, the ratio of non-symbiotic N fixation to the total N uptake by rice was between 19.6 and 23.0% in the three paddy soils in the Taihu Lake regions [23]. The average is 21.7%, equivalent to 11–16 kg N hm^−2^ of the BNF each season [23].

It has long been believed that photosynthetic bacteria (such as blue-green algae) and plant symbiotic bacteria (such as azolla and anabaena) are the main microorganisms responsible for BNF in the flooded paddy fields [25]. We recently found that the iron-reducing bacteria within Deltaproteobacteria can also fix N [26,27]. This group consists of two genera, *Anaeromyxobacter* and *Geobacter*, which are dominant microbial communities in the flooded paddy soils [26,27,28,29]. They usually use organic compounds such as acetic acid derived from straw decomposition as electron donors and Fe^3+^ as an electron acceptor for energy metabolism [30]. They may obtain electrons and transfer them to Fe^3+^ or ferric iron oxides through the respiratory chain and couple N fixation in this process when oxidizing organic compounds in anaerobic conditions [26,27,31]. Actually, in our previous study, the application of ferric iron oxides and rice straw to paddy soil microcosms enhanced the N-fixing activity of the soil, where *Anaeromyxobacter* and *Geobacter* might be involved in the enhancement of the soil N-fixing activity. In addition, the application of iron powder to paddy soil also enhanced the soil N-fixing activity [27]. Iron application also significantly enhanced the soil N-fixing activity of alfalfa, because iron is necessary in the synthesis of nitrogenase [32].

Accordingly, we hypothesize that reducing the N fertilization rate to paddy soil results in a reduction in rice yield, whereas iron powder application can alleviate the yield reduction, possibly through the enhancement of N-fixing activity of the iron-reducing bacteria. Further, a combination of less N fertilizer with iron powder ensures rice yield and simultaneously reduces the environmental N burden. We, therefore, conducted a 2-year field plot experiment to investigate the effects of such a combination on rice yield compared with conventional N fertilization. We also quantified the environmental N burden under different N fertilization rates alone or in combination with iron powder.

## 2. Materials and Methods

### 2.1. Field Site

The field site is located in Liuhe County, Nanjing, Jiangsu Province, China (118.69° E, 32.58° N), where the climate is a subtropical monsoon with around 254 frost-free days per year (Figure S1). The annual mean temperature and precipitation is 15.6 °C and 941.6 mm, respectively. The field site is a long-term traditional rice–wheat rotation land. The soil is a stagnic anthrosol [33,34]. Before the experiment began in June 2019, the soil at 0–20 °C m depth had a pH (H_2_O) of 5.9, total N and organic matter of 1.68 and 25.4 g kg^−1^ soil, and available P and K of 32 and 108 mg kg^−1^ soil, respectively.

### 2.2. Experimental Field and Design

Rice (*Oryza sativa* cv. Ninggeng 8) and wheat (*Triticum aestivum* cv. Zhenmai 168) were cultivated between 2019 and 2020 in twenty-eight 4 m × 5 m plots (Figure S1). Each plot was separated by a ridge of field compacted with soil. The rotation schedule was (1) rice from June to November and (2) wheat from November to June each year. Seven treatments were applied randomly, with four replicates of each; the treatments were 100%, 80%, 60%, and 0% of the conventional N fertilization rate, and 80%, 60%, and 0% of the conventional N with an iron amendment (Table 1). A pelleted poultry manure was applied basally at 1000 kg hm^−2^, except for the non-N treatment; this contained 7% of N, 3% of P_2_O_5_, 6% of K_2_O, and ≥20% of organic matter. The rest of the N in basal fertilizer was supplemented with urea. The supplementary fertilizer was urea only. During each rice-growing season, 60% of the N was applied basally before transplanting; 30% and 10% were used for supplementary fertilization at the tillering and jointing-booting stages, respectively. Calcium-magnesium phosphate (60 kg P_2_O_5_ hm^−2^), potassium chloride (105 kg K_2_O hm^−2^), and the pelleted poultry manure were applied, in addition to the urea, as basal fertilizers during each rice-growing season. Moreover, wheat or rice straw was incorporated into the soil at approximately 500 kg hm^−2^ or 1000 kg hm^−2^ before the cultivation of the next crop. Prior to the initiation of waterlogging in May 2019, the iron powder (zero-valent iron, >99% purity, Shijiazhuang, China) was applied to the soil surface at 5000 kg hm^−2^ and left to oxidize. The iron powder was applied only at the initial stage of the experiment in 2019. The irrigation, cultivation, and weed management practices were conventional, as used by local farmers. The paddy field was subjected to an intermittent irrigation regime. Briefly, the field surface was in a state of flood during the rice regreening stage, and dried under the sun in the late tillering stage. The field water was drained in the yellow maturity stage, and left under intermittent irrigation during the rest of the rice-growing stages. The rice yields were estimated by measuring the rice weight of each field plot.

### 2.3. Measurement of Soil Fe^2+^ Contents

Soil samples for Fe^2+^ content measurement were collected at 0–20 cm in the 2020 rice-growing season. Fe^2+^ content was measured spectrophotometrically with o-phenanthroline as a chromogenic reagent [35]. Briefly, 5.0 g of soil was passed through a 0.25 mm sieve and extracted with HCl solution. Then, 5 mL of the suspension were transferred into 50 mL of the volumetric flask and received 8 mL of a 100 g L^−1^ sodium acetate solution to adjust pH to 5.0. For color development, the chromogenic reagent was added as 10 mL of 1 g L^−1^ o-phenanthroline. Absorbance was measured with a spectrophotometer (UV754N, INESA (Group) Co., Ltd., Shanghai, China) at 510 nm after incubation for 30 min. Soil Fe^2+^ content was calculated using a standard curve made with (NH_4_)_2_Fe(SO_4_)_2_·6H_2_O.

### 2.4. NH_3_ Volatilization Measurement

A modified continuous airflow enclosure method measured the NH_3_ volatilization flux, which excluded plants in the chamber (20 cm diameter, 15 cm height) after the basal and supplementary fertilization [36,37]. The NH_3_ volatilization flux was determined twice daily, usually 7:00–9:00 and 14:00–16:00. In brief, the air was pumped by a vacuum pump at 15–20 chamber volumes min^−1^, then went through a tube into the NH_3_ absorbent (H_3_BO_3_ (2% *v:v*) plus mixed indicators of methyl red, bromocresol green, and ethanol). The measurements were conducted daily using the chemical titration method until the NH_3_ volatilization flux from each N fertilized plot was below the detection limit. The cumulative NH_3_ volatilization was calculated by the sum of daily NH_3_ emissions throughout the rice-growing season. The NH_3_ volatilization intensity was calculated by NH_3_ volatilization per unit of rice yield. Meanwhile, field surface water was collected from each field plot, and the NH_4_^+^ concentration and pH were determined spectrophotometrically or with a glass electrode (HI 2211, Hanna Instruments, Limena, Italy) [35].

### 2.5. N_2_O Emission Measurement

A closed chamber method evaluated the N_2_O flux from each field plot [37,38,39]. The N_2_O flux was measured every 3–5 days if higher than the background flux level and every 7–10 days if closer to the background flux level throughout the rice-growing season. In brief, each chamber base was tightly closed by a chamber box (0.5 m × 0.5 m × 0.6–1.2 m) with its height adjusted from 0.6 to 1.2 m depending on the heights of the plants. A gas sample was collected into a 15 mL vial every 15 min for a total period of 30 min (i.e., 0, 15, and 30 min); the temperature inside the chamber was also measured at each sampling. Soil moisture and temperature at a 10 cm depth were measured every hour with the Decagon 5TM volumetric water content and temperature sensor and recorded by a Meter ZL6 Advanced Cloud Data Logger (Meter Group, Inc., Pullman, Washington, DC, USA). The concentration of N_2_O was determined by a gas chromatograph with an electron capture detector (Agilent 7890B, Wilmington, DE, USA). N_2_O flux was generated by calculating the slope of N_2_O concentrations at three time points. The cumulative N_2_O emissions were calculated from each flux and the time between measurements.

### 2.6. NO_3_^−^ and NH_4_^+^ Leaching Measurement

A modified lysimetric method was used to collect NO_3_^−^ leaching samples in each field plot [37]. In brief, the lysimeter tube (10 cm diameter), made from 1 cm-thick hard plastic, was filled with quartz sand. The pipe wall had holes with a diameter of approximately 0.6 cm. After being wrapped with a nylon net, three lysimeters were embedded in the subsoils of each plot at a depth of 30, 60, and 90 cm (Figure S2). The leachate water was pumped through a soft plastic sampling tube connected to the lysimeters. The total volume of leachate water in the lysimeters was measured with a graduated cylinder, and the NO_3_^−^ and NH_4_^+^ concentrations were measured spectrophotometrically [35]. The leachate water was collected from the lysimeters every 2–3 weeks.

### 2.7. Statistical Analyses

Results were subjected to an analysis of variance (ANOVA) to determine the significance of the differences in data (means ± SD, *n* = 4) with SPSS 19.0 for Windows (IBM Corp., Armonk, NY, USA). Least significant difference (LSD) post hoc tests were performed to determine the differences between the individual treatments. Significant differences of means in all treatments were evaluated by LSD multiple comparison tests at the 5% level with SPSS 19.0 for Windows. A two-tailed significance test was performed to analyze the correlation between the NH_3_ volatilization flux and the NH_4_^+^ concentration and pH of field surface water.

## 3. Results

### 3.1. Soil Fe^2+^ Contents

**Figure 1:**
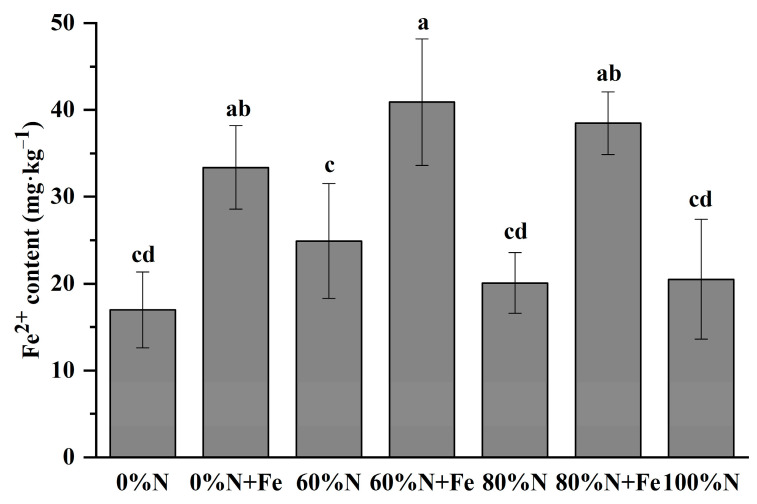
Fe^2+^ content of soil under various nitrogen fertilization rates with or without iron amendment in 2020. Conventional N fertilization rate (315 kg N hm^−2^ season^−1^) for rice in lower Yangtze River Delta is 100%N. Fe was applied at 5000 kg hm^−2^ as iron powder (>99% purity) when the field experiment was initiated. Treatments that have the same letter above their bars (means ± SD, *n* = 4) are not significantly different at *p* < 0.05 as determined by analysis of variance (one-way ANOVA), followed by least significance difference (LSD) post hoc test.

N fertilization had no significant effect on soil Fe^2+^ content (Figure 1). The iron application significantly increased soil Fe^2+^ content compared with the non-applied treatments, irrespective of the N fertilization rate.

### 3.2. Rice Yield

**Figure 2:**
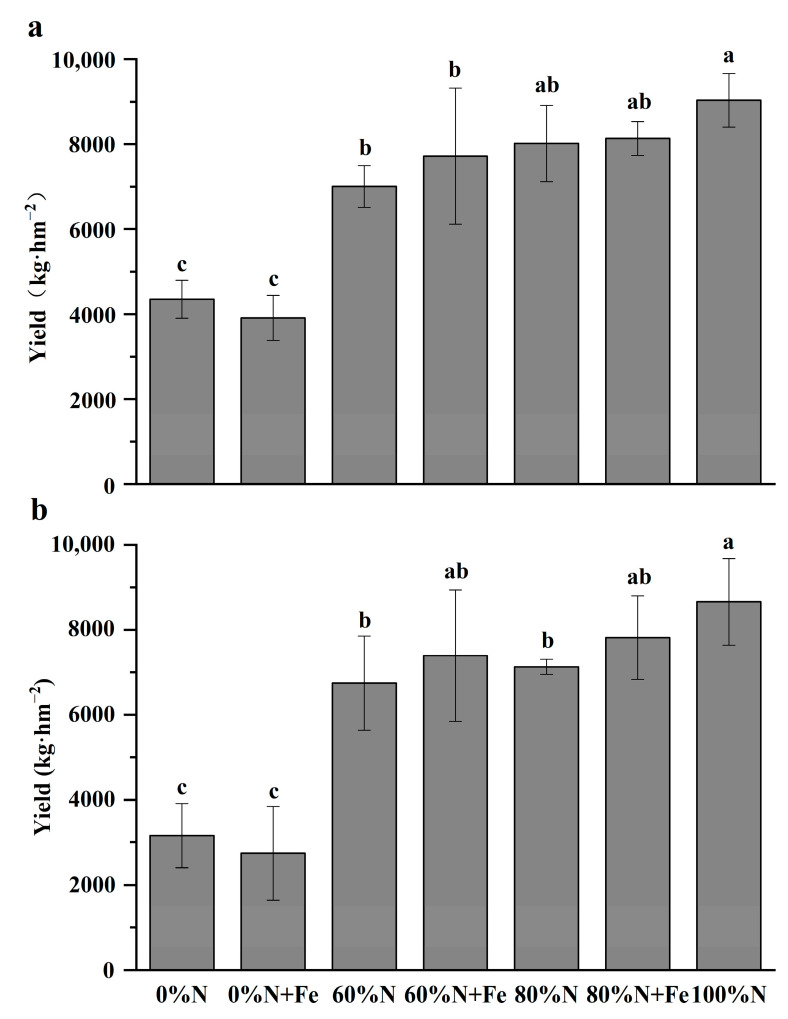
Rice yields under various nitrogen fertilization rates with or without iron amendment in 2019 (**a**) and 2020 (**b**). The conventional N fertilization rate (315 kg N hm^−2^ season^−1^) for rice in the lower Yangtze River Delta is 100%N. Fe was applied at 5000 kg hm^−2^ as iron powder (>99% purity) when the field experiment started. Treatments that have the same letter above their bars (means ± SD, *n* = 4) are not significantly different at *p* < 0.05 as determined by analysis of variance (one-way ANOVA), followed by least significance difference (LSD) post hoc test.

The rice yields decreased with a reduction in the conventional N fertilization rate (Figure 2). In the first rice-growing season (2019), the rice yields significantly decreased when there was a ≥40%N reduction in the conventional N fertilization rate, irrespective of the iron application (Figure 2a). In the second season, the yields significantly declined with the decreasing N fertilization rate when the iron powder was not applied (Figure 2b). However, the yields were comparable to the conventional N rate after the application of 80% and 60% of the conventional N rate in combination with iron powder for both seasons. During the 2020 season, we observed that 60%N + Fe supplementation resulted in a better rice yield than 80%N with no Fe supplementation. Moreover, we observed an increase of 9.7% and 9.6% after the iron application compared with the non-applied controls under these two N fertilization rates, respectively.

### 3.3. NH_3_ Volatilization

**Figure 3:**
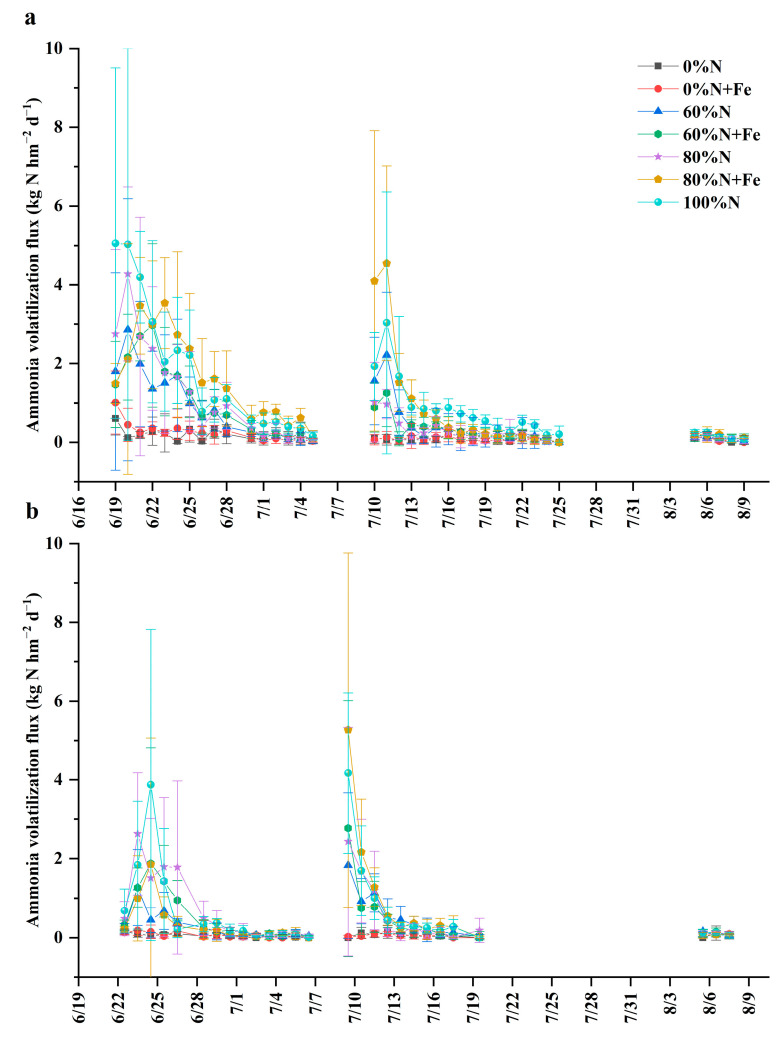
Ammonia volatilization flux from a paddy field under various nitrogen fertilization rates with or without iron amendment in 2019 (**a**) and 2020 (**b**). Conventional N fertilization rate (315 kg N hm^−2^ season^−1^) for rice in lower Yangtze River Delta is 100%N. Fe was applied at 5000 kg hm^−2^ as iron powder (>99% purity) when the field experiment was initiated.

The NH_3_ volatilization lasted for 10–14 days after the basal and first supplementary fertilization, whereas it lasted for 3–5 days after the second supplementary fertilization (Figure 3). The flux peaked 1–3 days after the basal and supplementary fertilization and then gradually declined to the background flux level. Overall, we observed that the NH_3_ volatilization flux decreased with a reduction in the conventional N fertilization rate. The flux peaks were more prominent under 80% of the conventional N plus the iron application than under the conventional N fertilization rate after the first supplementary fertilization. The flux was more pronounced in the first rice-growing season (Figure 3a) than in the second season (Figure 3b).

The cumulative NH_3_ volatilization and volatilization intensity generally decreased with a reduction in the conventional N fertilization rate (Table 2). They significantly decreased when the conventional N fertilization rate was reduced by ≤40%, irrespective of the iron application in the first rice-growing season. They were the least significant under the non-fertilized treatments while not significantly different among the N fertilized treatments in the second season. Moreover, no significant difference was observed between the iron application and the non-applied controls in both seasons. The cumulative NH_3_ volatilization and NH_3_ volatilization intensity were much lower in the second season than in the first season.

The NH_4_^+^ concentration in the field surface water notably increased after the N fertilization (Figure S3). It generally decreased with a reduction in the conventional N fertilization rate. It was greatest in the 80%N + Fe treatment after the second supplementary fertilization in the first rice-growing season (Figure S3a). The pH of the field surface water generally increased after the N fertilization (Figure S4). The NH_4_ ^+^ -N concentration (*r* = 0.682, *p* < 0.05) and pH (*r* = 0.302, *p* < 0.05) were significantly correlated with the NH_3_ volatilization flux, respectively.

### 3.4. N_2_O Emissions

**Figure 4:**
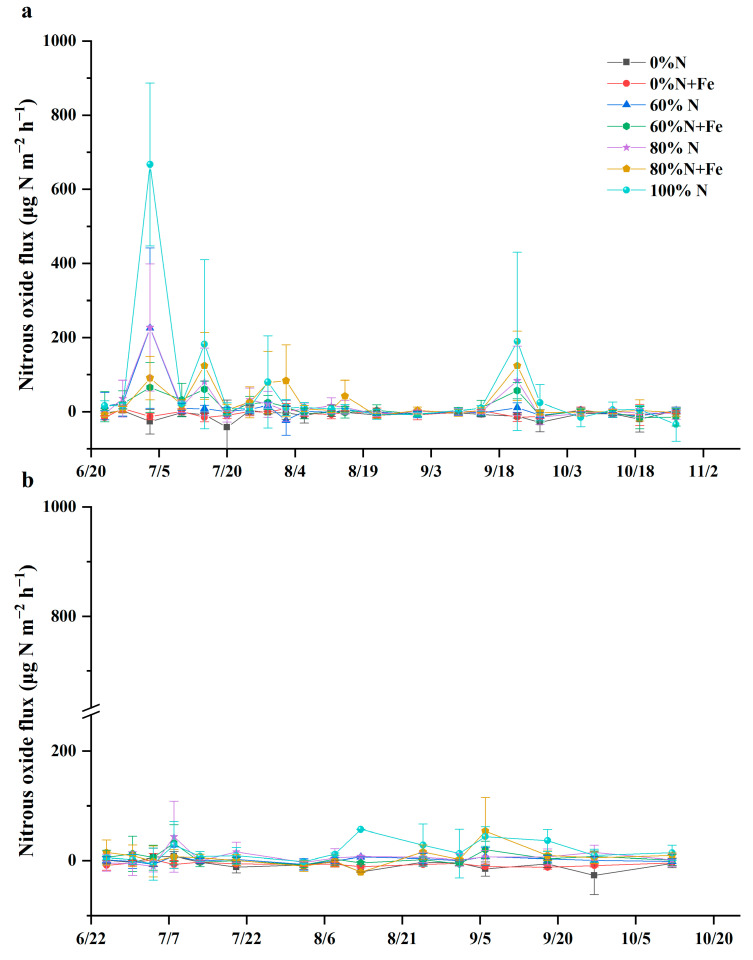
Nitrous oxide flux from a paddy under various nitrogen fertilization rates with or without iron amendment in 2019 (**a**) and 2020 (**b**). The conventional N fertilization rate (315 kg N hm^−2^ season^−1^) for rice in the lower Yangtze River Delta is 100%N. Fe was applied at 5000 kg hm^−2^ as iron powder (>99% purity) when the field experiment was initiated.

The pulse emission peaks were observed following the basal and supplementary fertilization (Figure 4). The N_2_O flux generally decreased with a reduction in the conventional N fertilization rate. In the first rice-growing season, the emission peaks were observed 15 days after the basal fertilization and 5 days after the supplementary fertilization (Figure 4a). Moreover, the last emission peak was observed at the drainage stage of the rice field, at which the soil moisture dropped (Figure S5). The emission peaks were observed 16 days after the basal fertilization and 10 days after the supplementary fertilization (Figure 4b).

In general, the cumulative N_2_O emissions and emission intensity decreased with a reduction in the conventional N fertilization rate, or was even negative under the 0%N treatment (Table 3). However, the cumulative N_2_O emissions decreased more rapidly than the reduction in the N fertilizer rate. Both N_2_O flux and cumulative emissions were much higher in the first rice-growing season than in the second season. The cumulative N_2_O emissions were generally higher after the iron application than the non-applied treatments under the same N fertilization rate. Further, it was significantly greater after the iron application in the 80%N treatment in the second rice-growing season.

### 3.5. NO_3_^−^ and NH_4_^+^ Leaching

In general, the NO_3_^−^ leaching decreased with a reduction in the conventional N fertilization rate or soil depth, whereas the NH_4_^+^ leaching fluctuated with the N fertilization rate and soil depth (Table 4 and Table 5). In the first rice-growing season, the NO_3_^−^ and NH_4_^+^ concentrations significantly increased after the iron application compared with the non-applied treatment under 80% of the conventional N rate at a depth of 30 cm (Table 4), as did the NO_3_^−^ concentration under 60% of the conventional N rate at a depth of 90 cm. The NO_3_^−^ concentration was generally higher in the second season than in the first season for the same treatments (Table 5). Moreover, it was comparable between the iron application and the non-applied treatments under the same N fertilization rate.

**Figure 5:**
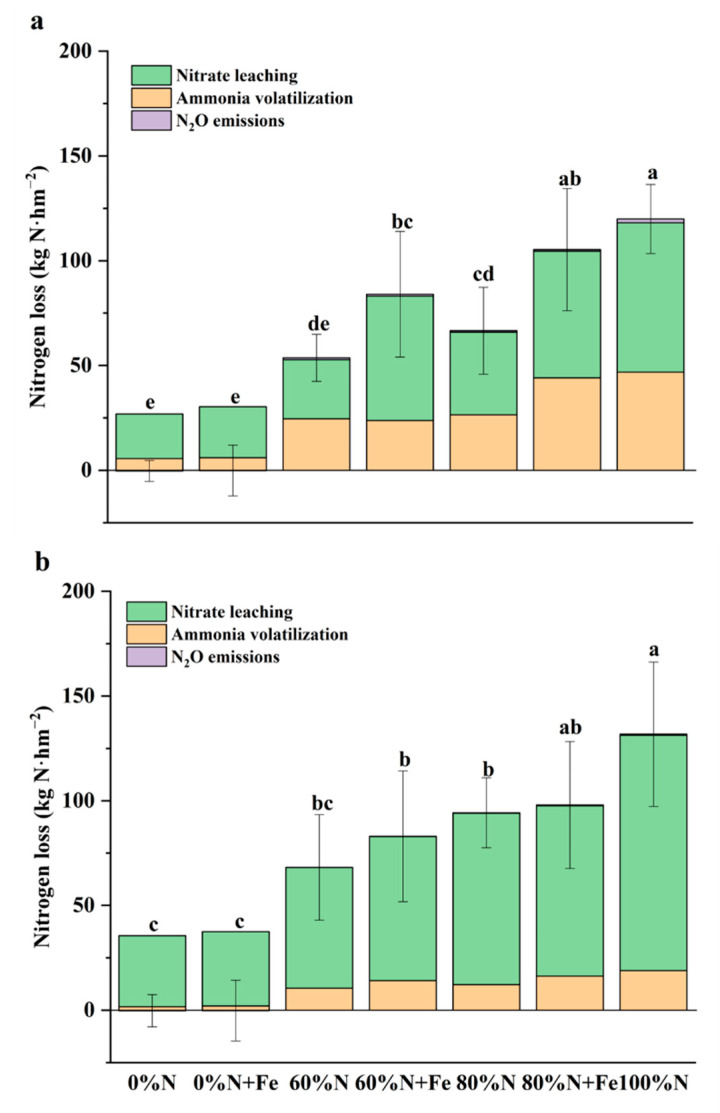
Nitrogen losses through ammonia volatilization, nitrous oxide emissions, and leaching N from a paddy field under various nitrogen fertilization rates with or without iron amendment in 2019 (**a**) and 2020 (**b**). the conventional N fertilization rate (315 kg N hm^−2^ season^−1^) for rice in the lower Yangtze River Delta is 100%N. Fe was applied at 5000 kg hm^−2^ as iron powder (>99% purity) when the field experiment was initiated. Treatments that have the same letter above their bars (means ± SD, *n* = 4) are not significantly different at *p* < 0.05 as determined by analysis of variance (one-way ANOVA), followed by least significance difference (LSD) post hoc test.

The cumulative N losses generally decreased with a reduction in the conventional N fertilization rate (Figure 5). Furthermore, the leaching N was the main N-loss pattern throughout the two rice-growing seasons. They accounted for 52.6–80.6% of the cumulated N losses in the first season and 82.9–95.9% in the second season. The NH_3_ volatilization accounted for 19.8–45.7% of the cumulative N losses in the first season and 4.8–17.0% in the second season. Moreover, the N_2_O emissions accounted for <2% of the cumulative N losses in the first season, and <1% in the second season.

## 4. Discussion

The present study showed that the N fertilization rate could be reduced with an iron amendment, probably through the enhancement of the N-fixing activity of the iron-reducing bacteria. Iron powder was applied to the soil surface at 5000 kg hm^−2^ and left to oxidize, which is equivalent to increasing the free iron oxide content in soil by 0.5% [27]. Soil nitrogen-fixing activity might be significantly enhanced by the addition of the generated ferric iron oxides to the paddy soil. The ferric iron oxides could provide electron acceptors for the iron-reducing bacteria, *Anaeromyxobacter* and *Geobacter*, to utilize for anaerobic respiration, thereby enhancing the soil nitrogen-fixing activity [27]. Moreover, a reduction in the conventional N fertilizer input decreased N losses from the field, such as NH_3_ volatilization, N_2_O emissions, and NO_3_^−^ leaching. This study may enable the establishment of novel and practical rice cultivation as a step towards green agriculture.

Studies have documented that BNF mostly accounted for the N fertility of the soil in the flooded paddy fields for thousands of years, before the use of synthetic fertilizers [18,23,24,40,41]. Significant measures were attempted to enhance BNF in the flooded paddy fields, including planting green manure crops before the rice-growing season and inoculating with Cyanobacteria, such as *Azolla* diazotrophic bacteria [42]. In this study, the iron amendment combined with crop straw is a feasible and straightforward method to enhance the BNF. It is critical to apply iron powder and straw and leave them for several days under the non-flooded condition to oxidize the iron to ferric iron oxides, since the ferric iron oxides such as ferrihydrite and lepidocrocite are electron acceptors in the energy metabolism of the iron-reducing bacteria [27,43]. Moreover, the acetic acid from the rice straw decomposition could serve as an electron donor for the iron-reducing bacteria [44]. An increase in soil Fe^2+^ content in iron powder-applied treatments compared with non-applied treatments suggests that the reduction of oxidized iron powder by iron-reducing bacteria and simultaneous N fixation probably occurred in the soil.

BNF usually decreases with an increase in N input, because high NH_4_^+^ concentration inhibits the growth and activity of diazotrophic *Anaeromyxobacter* [22,31]. Combining the iron amendment with 60–80% of the conventional N fertilization rate could ensure rice yields similar to those tested in this study, probably because the straw manure resulted in an increasing C:N ratio and alleviated the inhibition of high NH_4_^+^ on BNF. However, the iron amendment alone (0%N + Fe) could not increase rice yields, possibly because the current rice variety might have adapted to the nutrient-rich environment. No detrimental effects on wheat growth were observed between the rice crops in this two-year field experiment. Furthermore, the wheat yields in the 80%N + Fe treatment were comparable to the conventional N fertilization rate and increased by 9.7% compared with 80%N only in the third year of the field experiment.

The NH_3_ volatilization was reduced with a decrease in the N fertilization rate in this study, in agreement with the previous studies in flooded paddy fields [45,46,47]. Moreover, most NH_3_ volatilization occurred ≤3 days after the basal or supplementary fertilization [47]. In this study, the proportion of the NH_3_ volatilization to the applied N was 10.5–17.5% in the first rice-growing reason and 4.8–7.5% in the second season, which was higher than 1.4% in a Japanese paddy field [48]. Rice plants may be a channel for NH_3_ exchange between the soil and atmosphere, thereby underestimating the NH_3_ volatilization. In turn, dry deposition of NH_3_ is compensated to rice fields at ca. 7.3 kg N hm^−2^ year^−1^ [49].

The NH_4_^+^-N concentration in the field surface water (*r* = 0.682, *p* < 0.05) and pH (*r* = 0.302, *p* < 0.05) were significantly correlated with the NH_3_ volatilization flux. NH_3_ volatilization is influenced by several factors, in particular, N fertilization rate, pH, wind speed, NH_4_^+^-N concentration in the liquid phase, and temperature of the field surface water in the flooded paddy fields [45,47,50]. These factors generally show the following order in their strength: pH > wind speed > NH_4_^+^ concentration in liquid phase > temperature. The NH_3_ volatilization flux and cumulative NH_3_ volatilization were much lower in the second rice-growing season than in the first season, possibly because the rainfall was more frequent and lasted for longer periods. Prolonged rainfall and the N fertilization rate are crucial factors in determining NH_3_ volatilization loss [50]. Given the excessive longer rainfall periods and the varied N fertilization rates coupled with low pH, we can possibly interpret that these factors played a significant role in the loss of NH_3_ volatilization.

The N_2_O emissions were generally more pronounced after the iron amendment in the two rice-growing seasons and significantly higher under 80% of the conventional N fertilization rate in the second season. N_2_O emissions and Fe^2+^ were higher when N fertilization was supplemented with iron, probably because the iron oxides generated from iron powder dramatically increased the gene abundances of *nirS* and *nirK*, which encode nitrite reductase [11]. Moreover, the iron amendment might have enhanced the BNF of iron-reducing bacteria, and thus, enlarged the N pool of the paddy soils, thereby leading to N_2_O emissions from these soils via denitrification.

The N_2_O emissions were much lower in the second rice-growing season than in the first season, probably because the N_2_O reduction to N_2_ was enhanced under the strong reducing condition created by long and frequent rainfall. It also explains the lower N_2_O emissions during the early rice-growing period as compared to the middle and late period of the second season. The paddy soils were N_2_O sources and sinks, particularly under anaerobic conditions [51]. Long and frequent rainfall resulted in higher soil moisture in the second season than in the first season (Figure S5), which could enhance the N_2_O reduction to N_2_ in strong reductive conditions.

The NO_3_^−^ was the predominant N form of the total leached inorganic N in these constant charge paddy soils [52,53], which was notably decreased with a decrease in the N fertilization rate. The NO_3_^−^ leaching was much more significant in the second rice-growing season than in the first season because of frequent and more prolonged rainfall. However, this contribution may be overestimated because the total leaching losses were the sum of leaching losses at a depth of 30, 60, and 90 cm. The plants probably utilized NH_4_^+^ and NO_3_^−^ leaching at a depth of 30 and 60 cm due to the upward migration of water [54]. Overall, the cumulative N losses measured in this study decreased with a reduction in the conventional N fertilization rate.

The field site was located in the lower Yangtze River Delta, where the rice originated and has been cultivated for thousands of years. Long-term ecological experiments are an important tool to clarify the effects of iron powder applied with less N fertilizer on rice yields and critical N losses from paddy fields. Therefore, our field experiment is expected to be maintained for a longer term to confirm these effects in China’s lower Yangtze River Delta. Further field experiments may be constructed to investigate the effects of iron powder applied with less N fertilizer in the main paddy soils across China and Japan.

## 5. Conclusions

The iron powder application at 5000 kg hm^−2^ when the field experiment was initiated ensured rice yields with a 20–40% reduction in the conventional N fertilization level of ca. 315 kg N hm^−2^ in a paddy field in China’s Yangtze River Delta. The rice yields were significantly decreased with a 20–40% reduction in the conventional N fertilization rate with the treatment time extended, whereas they increased by 9.7% and 9.6% after the iron application relative to the non-applied controls. Furthermore, the measured N losses, such as NH_3_ volatilization, N_2_O emissions, and NO_3_^−^ leaching, were generally minimized due to the reduction in the conventional N fertilization rate. The N losses were notable with regard to NO_3_^−^ leaching, higher with regard to NH_3_ volatilization, and the least with regard to N_2_O emissions. Combining the iron application with a 20–40% reduction in the conventional N fertilization rate could maintain rice yields while reducing the critical N losses from the flooded paddy soil in China’s Yangtze River Delta. Our study leads to the establishment of novel and practical rice cultivation as a step towards green agriculture in this area.

## Figures and Tables

**Table 1:** Treatments and fertilization rate in the field plots.

Treatment	Wheat (kg N hm^−2^)		Rice (kg N hm^−2^)		
	Basal Fertilizer	Supplementary Fertilizer	Basal Fertilizer	First Supplementary Fertilizer	Second Supplementary Fertilizer
0%N	0	0	0	0	0
0%N + Fe	0	0	0	0	0
60%N	89.1	59.4	113.4	56.7	18.9
60%N + Fe	89.1	59.4	113.4	56.7	18.9
80%N	118.8	79.2	151.2	75.6	25.2
80%N + Fe	118.8	79.2	151.2	75.6	25.2
100%N	148.5	99	189	94.5	31.5

A commercial organic manure was applied basally at 1000 kg hm^−2^, except for the non-N treatment, which contained 7% of N, 3% of P_2_O_5_, 6% of K_2_O, and ≥20% of organic matter. The rest of the N in basal fertilizer was supplemented with urea. The supplementary fertilizer was urea only. All treatments received 67.5 kg P_2_O_5_ hm^−2^ of calcium-magnesium phosphate and 76.5 kg K_2_O hm^−2^ of potassium chloride as basal fertilizers in each wheat-growing season, and received 60 kg P_2_O_5_ hm^−2^ of calcium-magnesium phosphate and 105 kg K_2_O hm^−2^ of potassium chloride as basal fertilizers in each rice-growing season. The iron powder (>99% purity) was applied at 5000 kg hm^−2^ only at the initial stage of the experiment in 2019.

**Table 2:** Cumulative ammonia volatilization and ammonia volatilization intensity under various nitrogen fertilization rates with or without iron amendment in two rice-growing seasons.

Treatment	Cumulative Ammonia Volatilization (kg N hm^−2^)		Ammonia Volatilization Intensity (g N kg^−1^)	
	2019	2020	2019	2020
0%N	5.6 ± 1.8 c	1.7 ± 0.5 b	1.3 ± 0.4 c	0.6 ± 0.2 c
0%N + Fe	6.0 ± 1.6 c	2.0 ± 0.5 b	1.5 ± 0.3 c	0.8 ± 0.4 bc
60%N	22.1 ± 13.0 b	10.5 ± 3.3 ab	3.6 ± 1.8 b	1.6 ± 0.6 abc
60%N + Fe	23.8 ± 4.3 b	14.1 ± 6.1 a	3.1 ± 0.5 b	2.0 ± 0.9 ab
80%N	26.5 ± 11.0 b	12.2 ± 1.5 a	3.3 ± 1.1 b	2.2 ± 1.5 a
80%N + Fe	44.1 ± 9.7 a	16.3 ± 8.1 a	5.5 ± 1.4 a	2.1 ± 1.2 a
100%N	46.8 ± 9.7 a	18.9 ± 7.4 a	5.2 ± 0.8 a	2.1 ± 0.6 a

The conventional N fertilization rate (315 kg N hm^−2^ season^−1^) for rice in the lower Yangtze River Delta is 100%N. Fe was applied at 5000 kg hm^−2^ as iron powder (>99% purity) when the field experiment began. Values (means ± SD, *n* = 4) followed by the same letter in columns are not significantly different at *p* < 0.05 as determined by analysis of variance (one-way ANOVA), followed by least significance difference (LSD) post hoc test.

**Table 3:** Cumulative nitrous oxide (N_2_O) emissions and N_2_O emission intensity under various nitrogen fertilization rates with or without iron amendment in two rice-growing seasons.

Treatment	Cumulative N_2_O Emissions (g N hm^−2^)		N_2_O Emission Intensity (kg CO_2_-eq·kg^−1^)	
	2019	2020	2019	2020
0%N	−271.97 ± 110.73 d	−232.56 ± 87.90 e	−0.02 ± 0.01 d	−0.02 ± 0.01 c
0%N + Fe	−122.8 ± 70.06 cd	−194.85 ± 13.98 e	−0.01 ± 0.00 cd	−0.02 ± 0.01 c
60%N	353.26 ± 352.78 bcd	27.92 ± 62.90 d	0.01 ± 0.01 bc	0.00 ± 0.00 b
60%N + Fe	386.60 ± 140.08 bc	92.30 ± 80.82 d	0.01 ± 0.01 bc	0.00 ± 0.00 b
80%N	659.25 ± 510.83 b	207.74 ± 55.13 c	0.02 ± 0.02 b	0.01 ± 0.00 b
80%N + Fe	760.79 ± 573.42 b	343.53 ± 76.68 b	0.03 ± 0.02 b	0.01 ± 0.01 b
100%N	1737.08 ± 722.16 a	472.14 ± 124.40 a	0.05 ± 0.02 a	0.01 ± 0.01 a

The conventional N fertilization rate (315 kg N hm^−2^ season^−1^) for rice in the lower Yangtze River Delta is 100%N. Fe was applied at 5000 kg hm^−2^ as reduced iron powder (99%) when the field experiment began. Values (means ± SD, *n* = 4) followed by the same letter in columns are not significantly different at *p* < 0.05 as determined by analysis of variance (one-way ANOVA), followed by least significance difference (LSD) post hoc test.

**Table 4:** Ammonium and nitrate leaching from a paddy field at a soil depth of 30, 60, and 90 cm under various nitrogen fertilization rates with or without iron amendment in 2019.

Treatment	NO_3_^−^-N (kg·hm^−2^)			NH_4_^+^-N (kg·hm^−2^)		
	30 cm	60 cm	90 cm	30 cm	60 cm	90 cm
0%N	10.60 ± 3.53 b	6.93 ± 2.93 b	3.76 ± 1.09 b	1.99 ± 1.79 b	0.77 ± 0.40 b	−0.11 ± 0.09 b
0%N + Fe	13.13 ± 7.74 b	8.68 ± 3.10 b	2.53 ± 0.53 b	1.44 ± 3.43 b	1.43 ± 1.80 b	0.13 ± 0.02 b
60%N	14.08 ± 7.03 b	7.80 ± 3.11 b	6.37 ± 3.15 b	0.29 ± 0.70 b	2.60 ± 1.98 ab	1.78 ± 3.31 b
60%N + Fe	21.07 ± 4.95 ab	18.20 ± 12.00 ab	20.16 ± 14.27 a	3.17 ± 2.68 ab	7.05 ± 5.89 a	3.10 ± 3.36 b
80%N	16.94 ± 6.36 b	14.11 ± 7.34 ab	8.37 ± 2.28 b	0.48 ± 1.98 b	4.20 ± 3.73 ab	3.63 ± 2.77 b
80%N + Fe	30.83 ± 7.83 a	21.62 ± 13.69 a	8.03 ± 4.78 b	12.01 ± 11.64 a	2.86 ± 3.59 ab	1.22 ± 1.86 b
100%N	31.93 ± 14.32 a	17.28 ± 4.11 ab	22.11 ± 8.11 a	7.90 ± 10.78 ab	0.29 ± 0.63 b	7.92 ± 3.85 a

The conventional N fertilization rate (315 kg N hm^−2^ season^−1^) for rice in the lower Yangtze River Delta is 100%N. Fe was applied at 5000 kg hm^−2^ as reduced iron powder (99%) when the field experiment began. Values (means ± SD, *n* = 4) followed by the same letter in columns are not significantly different at *p* < 0.05 as determined by analysis of variance (one-way ANOVA), followed by least significance difference (LSD) post hoc test.

**Table 5:** Ammonium and nitrate leaching from a paddy field at a soil depth of 30, 60, and 90 cm under various nitrogen fertilization rates with or without iron amendment in 2020.

Treatment	NO_3_^−^-N (kg hm^−2^)			NH_4_^+^-N (kg hm^−2^)		
	30 cm	60 cm	90 cm	30 cm	60 cm	90 cm
0%N	14.71 ± 2.62 c	10.80 ± 7.40 b	8.37 ± 3.78 c	0.30 ± 0.12 b	0.26 ± 0.15 b	0.13 ± 0.21 b
0%N + Fe	14.32 ± 7.25 c	15.89 ± 11.50 b	5.29 ± 2.56 c	0.43 ± 0.08 b	0.62 ± 0.66 ab	0.13 ± 0.07 b
60%N	25.09 ± 10.53 bc	20.18 ± 12.97 b	12.39 ± 6.01 bc	3.55 ± 1.83 a	0.91 ± 0.65 ab	0.11 ± 0.04 b
60%N + Fe	28.02 ± 15.45 abc	27.85 ± 10.82 ab	12.93 ± 9.48 bc	0.97 ± 1.34 b	1.56 ± 1.49 a	0.34 ± 0.47 b
80%N	30.49 ± 2.01 abc	27.81 ± 8.14 ab	23.56 ± 11.05 ab	1.67 ± 1.02 b	1.08 ± 1.00 ab	0.51 ± 0.59 ab
80%N + Fe	33.01 ± 10.01 ab	21.09 ± 4.52 b	27.25 ± 17.52 a	1.99 ± 1.61 ab	1.01 ± 0.66 ab	0.55 ± 0.79 ab
100%N	44.77 ± 22.63 a	48.26 ± 28.54 a	19.33 ± 10.91 abc	0.98 ± 0.71 b	0.67 ± 0.89 ab	1.33 ± 1.33 a

The conventional N fertilization rate (315 kg N hm^−2^ season^−1^) for rice in the lower Yangtze River Delta is 100%N. Fe was applied at 5000 kg hm^−2^ as reduced iron powder (99%) when the field experiment began. Values (means ± SD, *n* = 4) followed by the same letter in columns are not significantly different at *p* < 0.05 as determined by analysis of variance (one-way ANOVA), followed by least significance difference (LSD) post hoc test.

## Data Availability

Not applicable.

## Supplementary Materials

The following are available online at https://www.mdpi.com/article/10.3390/ijerph19148707/s1, Figure S1: Field experiment testing rice yields and critical nitrogen loss from flooded paddy fields under various nitrogen fertilization rates with or without iron amendment, Figure S2: Leachate collection device to collect NO_3_^−^ leaching samples in each field plot, Figure S3: Ammonia concentration in field surface water under various nitrogen fertilization rates with or without iron amendment in 2019 (a) and 2020 (b), Figure S4: PH of field surface water under various nitrogen fertilization rates with or without iron amendment in 2019 (a) and 2020 (b), Figure S5: Soil moisture and temperature in the experimental field in 2019 (a) and 2020 (b).
