# Supplementary material for: Investigation of Rice Yields and Critical N Losses from Paddy Soil under Different N Fertilization Rates with Iron Application
Source: Int J Environ Res Public Health. 2022 Jul 17;19(14):8707. doi: 10.3390/ijerph19148707 (PMC9318169; doi:10.3390/ijerph19148707)
Supplement: Supplementary file 1 [file ijerph-19-08707-s001.zip › ijerph-1780893-supplementary.pdf]

**Figure S1.** Field experiment testing rice yields and critical nitrogen loss from flooded paddy fields under various nitrogen fertilization rates with or without iron amendment.

**Figure S2.** Leachate collection device to collect $NO_3^-$ leaching samples in each field plot.

**Figure S3.** Ammonia concentration in field surface water under various nitrogen fertilization rates with or without iron amendment in 2019 (**a**) and 2020 (**b**). 100%N is the conventional N fertilization rate (315 kg N $hm^{-2}$ $season^{-1}$) for rice in the lower Yangtze River Delta. Fe was applied at 5000 kg $hm^{-2}$ of iron powder (>99% purity) started the beginning of the field experiment.

**Figure S4.** PH of field surface water under various nitrogen fertilization rates with or without iron amendment in 2019 (**a**) and 2020 (**b**). 100%N is the conventional N fertilization rate (315 kg N $hm^{-2}$ $season^{-1}$) for rice in the lower Yangtze River Delta. Fe was applied at 5000 kg $hm^{-2}$ of iron powder (>99% purity) started the beginning of the field experiment.

**Figure S5.** Soil moisture and temperature in the experimental field in 2019 (**a**) and 2020 (**b**).

**Figure S1**

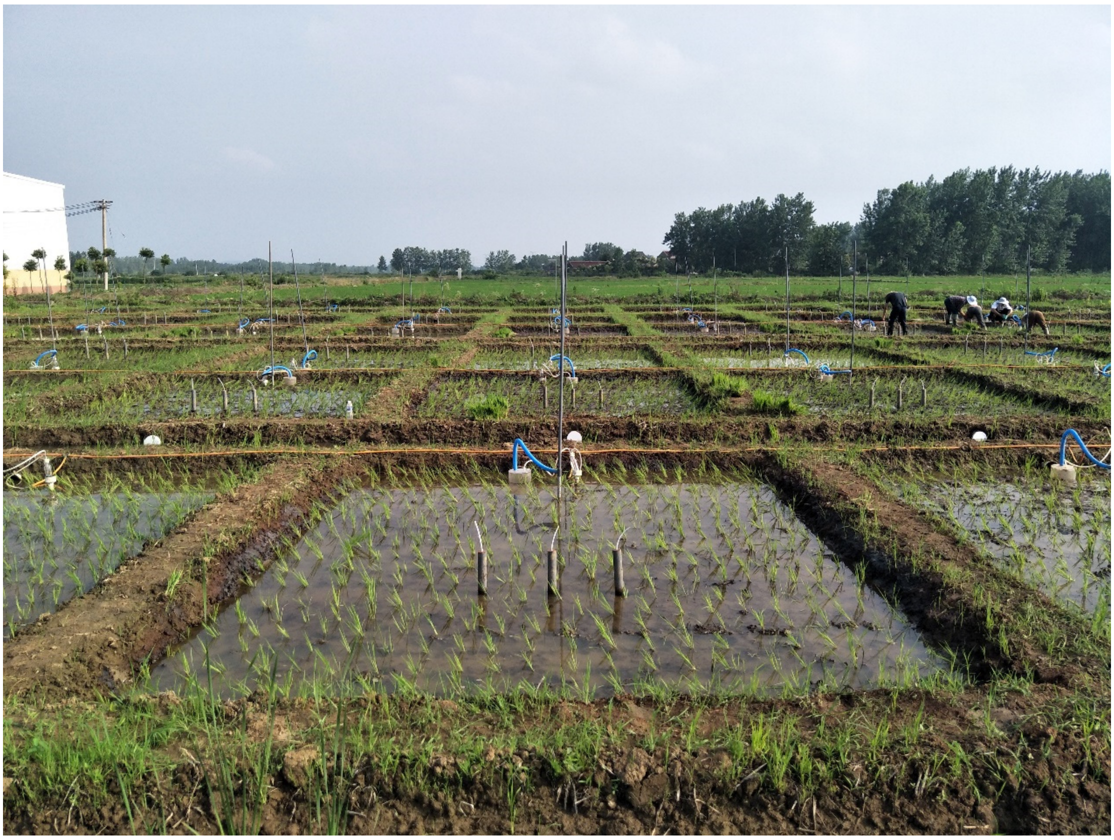

**Figure S2**

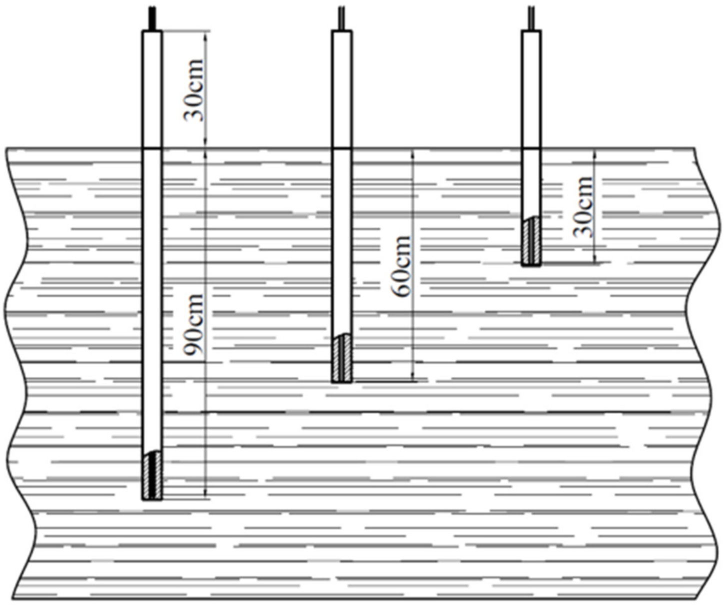

## Figure S3

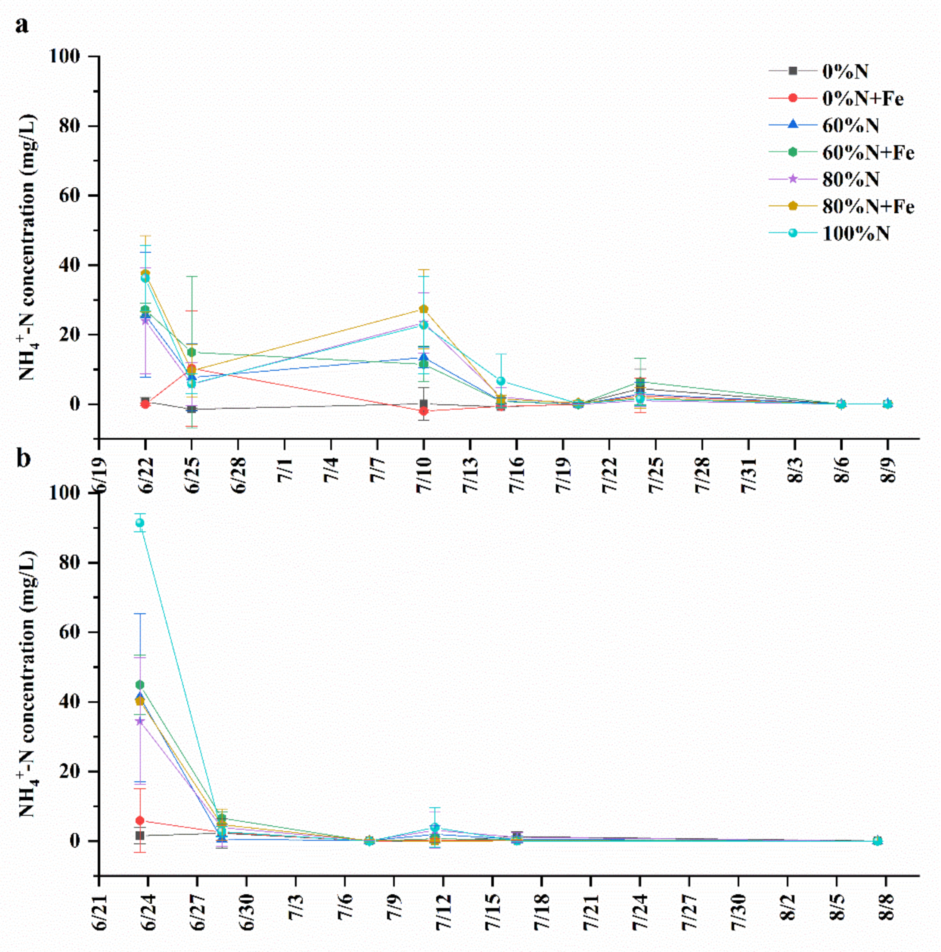
a
b
NH4+-N concentration (mg/L)
0%N
0%N+Fe
60%N
60%N+Fe
80%N
80%N+Fe
100%N
6/19
6/22
6/25
6/28
7/1
7/4
7/7
7/10
7/13
7/16
7/19
7/22
7/25
7/28
7/31
8/3
8/6
8/9
6/21
6/24
6/27
6/30
7/3
7/6
7/9
7/12
7/15
7/18
7/21
7/24
7/27
7/30
8/2
8/5
8/8

## Figure S4

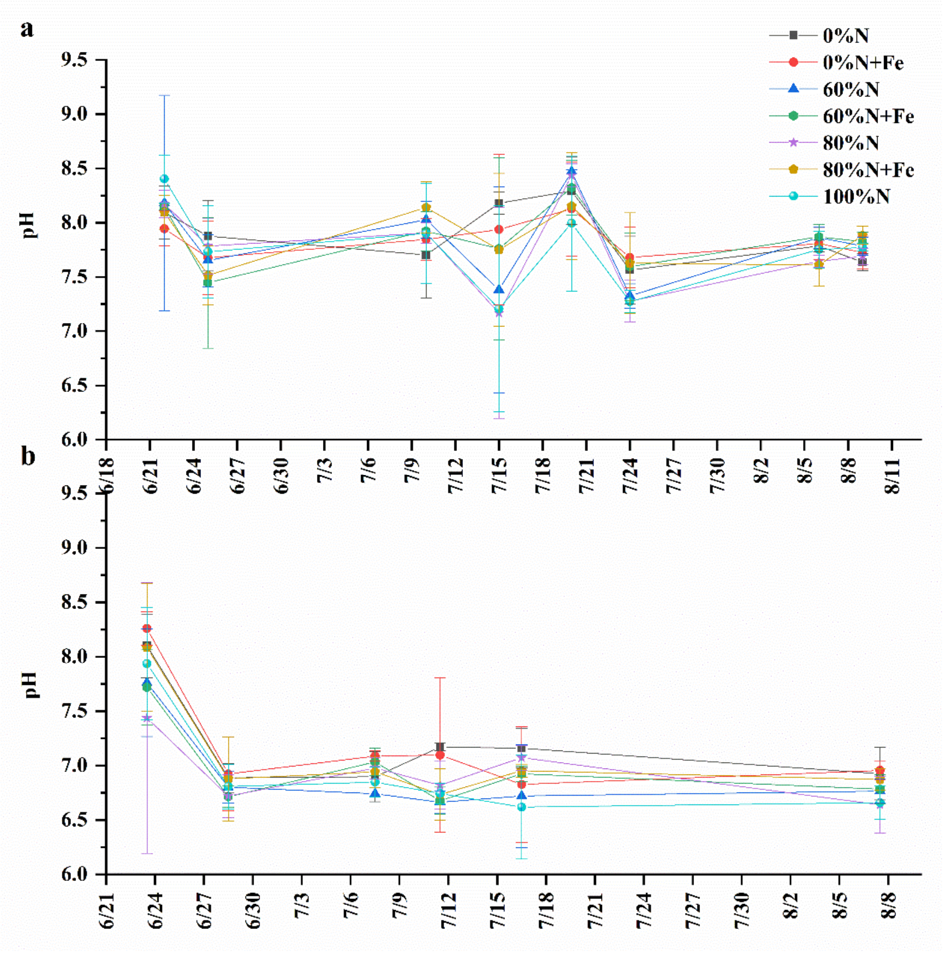

a
b
pH
pH
0%N
0%N+Fe
60%N
60%N+Fe
80%N
80%N+Fe
100%N
9.5
9.0
8.5
8.0
7.5
7.0
6.5
6.0
6/18
6/21
6/24
6/27
6/30
7/3
7/6
7/9
7/12
7/15
7/18
7/21
7/24
7/27
7/30
8/2
8/5
8/8
8/11

**Figure S5**

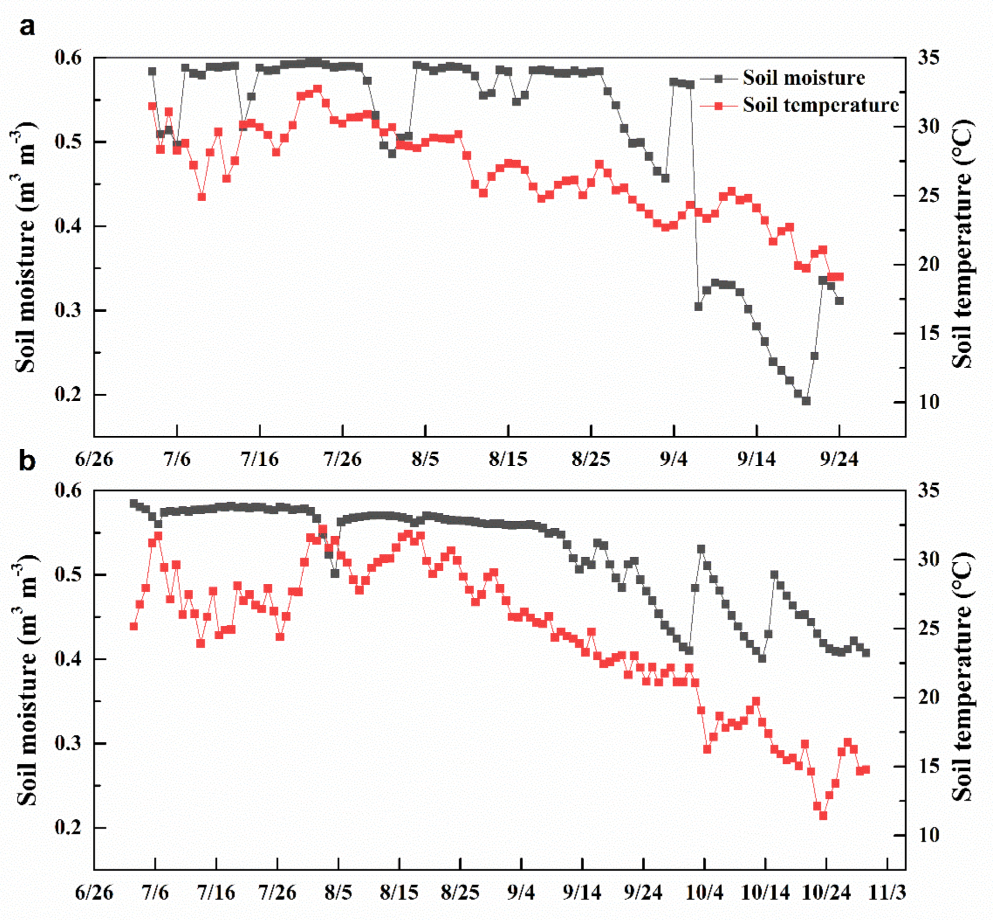

a
Soil moisture ($m^3$ $m^{-3}$)
Soil temperature (°C)
Soil moisture
Soil temperature
0.6
0.5
0.4
0.3
0.2
35
30
25
20
15
10
6/26
7/6
7/16
7/26
8/5
8/15
8/25
9/4
9/14
9/24
b
Soil moisture ($m^3$ $m^{-3}$)
Soil temperature (°C)
0.6
0.5
0.4
0.3
0.2
35
30
25
20
15
10
6/26
7/6
7/16
7/26
8/5
8/15
8/25
9/4
9/14
9/24
10/4
10/14
10/24
11/3
